# A Simplified Technique for Glaucoma Drainage Device Ciliary Sulcus Implantation

**DOI:** 10.7759/cureus.105123

**Published:** 2026-03-12

**Authors:** Zaid Alsafi, Sana Hamid, Hardeep Kandola, Ahmed Elkarmouty

**Affiliations:** 1 Glaucoma Department, Moorfields Eye Hospital, London, GBR

**Keywords:** glaucoma drainage device, glaucoma drainage implants, glaucoma practice, primary open angle glaucoma, secondary glaucoma

## Abstract

Purpose

The study aimed to describe a simplified technique for ciliary sulcus (CS) placement of glaucoma drainage devices (GDD) and to report long-term clinical outcomes in phakic and pseudophakic eyes.

Methods

This retrospective case series included 10 eyes of 10 patients who underwent CS placement of a PAUL Glaucoma Implant (Advanced Ophthalmic Innovations, Singapore) or Baerveldt 350 mm² implant (Advanced Medical Optics, Santa Ana, USA) at a large tertiary referral eye unit in London in 2021. Indications included uncontrolled intraocular pressure (IOP) despite maximal medical therapy and prior unsuccessful glaucoma surgery, or a low likelihood of trabeculectomy success. Pre- and postoperative data collected by case note review included IOP, visual acuity, visual field indices, number of IOP-lowering medications, and complications. A standardised technique using a 90-degree bent needle to create a controlled scleral tunnel directing the tube into the CS was employed in all cases.

Results

Mean follow-up was 47.7 months (range 36-57 months). Mean IOP decreased significantly from 28.7 ± 6.9 mmHg preoperatively to 14.4 ± 2.9 mmHg at final follow-up (p = 0.002). The median number of IOP-lowering medications decreased from three preoperatively to 1.5 at final follow-up (p = 0.014). Visual acuity (logMAR) and visual field mean deviation remained stable over follow-up. Six eyes required stent suture removal, and one eye underwent early paracentesis for suspected tube obstruction. Additional glaucoma surgery was required in three pseudophakic eyes with prior glaucoma surgery. One eye developed corneal decompensation requiring a graft; this eye had chronic uveitis and extensive anterior synechiae, and neither the anterior chamber nor sulcus tube was in contact with the corneal endothelium on serial examination. Eyes without prior glaucoma surgery experienced fewer postoperative events.

Conclusions

Ciliary sulcus placement of GDD tubes using a simplified entry technique achieved durable IOP reduction with preservation of visual function and an acceptable safety profile over long-term follow-up. The technique appears particularly useful in eyes at increased risk of corneal complications or without a suitable anterior chamber and provides a reproducible alternative to anterior chamber tube placement in both phakic and pseudophakic patients.

## Introduction

Glaucoma drainage devices (GDD) are widely utilised in the surgical management of persistently raised intraocular pressure (IOP). A major complication associated with GDDs is corneal endothelial cell loss when compared to trabeculectomy [1]. Over five years in the Tube vs Trabeculectomy (TVT) Study, persistent corneal oedema occurred in 16% of Baerveldt glaucoma implant (Advanced Medical Optics, Santa Ana, USA) eyes and 9% of trabeculectomy eyes, with keratoplasty ultimately required in six tube cases and five trabeculectomy cases [2]. Additionally, in a systematic review of PAUL Glaucoma Implant (Advanced Ophthalmic Innovations, Singapore) outcomes, Tan et al. reported a pooled corneal decompensation rate of 4.1% across studies, with a mean follow-up ranging from 2 to 35.8 months [3]. Corneal decompensation is more common when there is cornea-tube touch, in tubes entering the anterior chamber (AC) proximal to Schwalbe’s line and in mispositioned tubes, which may intermittently rub against the endothelium, including during eye rubbing, eye movements and blinking [4]. With AC placement of Baerveldt tubes, increasing the distance between GDD and the corneal surface reduces endothelial cell density (ECD) loss [5]. Loss of ECD is often greatest at the cornea closest to the tube [4]. As well as causing corneal decompensation, AC placement can also result in blockage of the GDD, cataractogenesis, and iritis [6].

A healthy adult cornea typically has an ECD of 2,000-3,000 cells/mm². Clinical observational studies indicate that eyes with endothelial cell densities in the range of 500-800 cells/mm² are at high risk of endothelial failure and corneal oedema, particularly following intraocular surgery [7]. To reduce these complications, ciliary sulcus (CS) placement can be performed. CS placement is useful in pseudophakic or aphakic eyes or in those with shallow anterior chambers and peripheral anterior synechiae, as well as in patients with known compromised corneal endothelial function or corneal grafts [8]. Zhang et al. demonstrated that the mean monthly central ECD loss for AC placement is almost double that of CS at 29.33 and 15.32 cells/mm², respectively [9]. For comparison, under normal physiological conditions, corneal endothelial cell density decreases gradually with age, at an average rate of 1-2 cells/mm² per month in healthy adults and of 7.98 cells/mm² per month over 36 months, following phacoemulsification [10,11].

In this retrospective case series, we describe a simplified technique for GDD implantation into the CS in phakic and pseudophakic patients and evaluate the long-term clinical outcomes associated with this technique.

## Materials and methods

All procedures were performed by a single surgeon at a large tertiary referral eye unit in London, United Kingdom, on 10 eyes of 10 patients in 2021. Written informed consent was obtained from all patients after a detailed explanation of the nature of the procedure, associated risks, and potential adverse outcomes. Patients were selected for glaucoma drainage device (GDD) surgery based on uncontrolled intraocular pressure despite maximally tolerated medical therapy, prior laser trabeculoplasty, and at least one unsuccessful trabeculectomy or GDD. In four cases, GDD implantation was undertaken as the primary surgical intervention due to a low anticipated likelihood of trabeculectomy success based on the underlying glaucoma subtype. Preoperative and postoperative data were collected for each eye through retrospective review of medical records and included visual acuity, intraocular pressure, number of pressure-lowering medications, and intraoperative and postoperative complications. Institutional approval was obtained to undertake this retrospective audit. Patient consent was obtained to record and publish the procedures.

Statistical analysis

Descriptive statistics were used to summarise the data using GraphPad Prism 9.5.1. for Windows (GraphPad Software, Boston, USA). Normality was assessed using the Shapiro-Wilk test, and a p-value < 0.05 was considered statistically significant.

Surgical technique

Full details of the materials and equipment used are provided in the Appendices. A PAUL Glaucoma Implant (PGI; Advanced Ophthalmic Innovations, Singapore) was used in six cases, and a Baerveldt 350 mm² implant (BVT-350; Advanced Medical Optics, Santa Ana, USA) was used in four cases. The surgical technique is depicted in Video 1 and Video 2.

**Video 1:** Surgical Technique for Ciliary Sulcus Placement of a Glaucoma Drainage Device – Example 1 Surgical technique for ciliary sulcus placement of a glaucoma drainage device in a patient with a prior deep anterior lamellar keratoplasty for corneal ectasia. Following sub-Tenon’s anaesthesia and superotemporal peritomy, the implant plate is secured beneath the rectus muscles with adjunctive mitomycin C. The tube is trimmed bevel-up and introduced through a short scleral tunnel created with a 90-degree bent needle, directing entry into the ciliary sulcus while minimising the risk of anterior misdirection. The tube is advanced under direct control using a ripcord stent to maintain rigidity, positioned to avoid iris contact, and secured to the sclera before patch graft and conjunctival closure.

**Video 2:** Surgical Technique for Ciliary Sulcus Placement of a Glaucoma Drainage Device – Example 2 Surgical technique for ciliary sulcus placement of a glaucoma drainage device in a phakic patient without prior surgical intervention. Following sub-Tenon’s anaesthesia and superotemporal peritomy, the implant plate is secured beneath the rectus muscles with adjunctive mitomycin C. The tube is trimmed bevel-up and introduced through a short scleral tunnel created with a 90-degree bent needle, directing entry into the ciliary sulcus while minimising the risk of anterior misdirection. The tube is advanced under direct control using a ripcord stent to maintain rigidity, positioned to avoid iris contact, and secured to the sclera before patch graft and conjunctival closure.

All procedures were performed under local anaesthesia with a sub-Tenon’s injection of lidocaine combined with bupivacaine. A superotemporal conjunctival peritomy was fashioned, and the space between Tenon’s capsule and sclera was dissected. Mitomycin C (0.4 mg/mL) was applied on surgical sponges for 5 minutes and subsequently irrigated with 30 mL of balanced salt solution. After priming the implant with balanced salt solution, a 6-0 Prolene suture (Johnson & Johnson MedTech Ltd, Wokingham, United Kingdom) or Supramid suture (Advanced Medical Solutions, Winsford, United Kingdom) was used to stent the tube for the PGI and BVT-350, respectively. The lateral and superior rectus muscles were isolated using muscle hooks, and the plate wings were positioned beneath the muscles and secured to the underlying sclera with a 9-0 Prolene suture.

The globe was returned to the primary position. Using callipers, a point 2 mm posterior to the surgical limbus was marked in an imaginary plane aligned with the centre of the pupil. The tube was trimmed in a bevel-up configuration to achieve an intraocular length of approximately 4-5 mm, ensuring that the tube remained visible beneath the iris margin on pupillary dilation.

A scleral tunnel was created using a 25-gauge needle for the PGI or a 23-gauge needle for the BVT-350 to facilitate passage of the tube into the ciliary sulcus. The needle tip was bent to approximately 90 degrees just posterior to the bevel. The needle was inserted just posterior to the marked entry site at an acute angle to create a short scleral tunnel before being directed downward to enter the anterior chamber.

Once approximately half of the bevel had entered the eye, the needle tip was elevated and advanced forward. This manoeuvre tents the iris and creates a short tunnel directing the tube toward the ciliary sulcus. The 90-degree bend in the needle acts as a safety guard against inadvertent anterior misdirection, thereby reducing the risk of lens injury.

The tube was introduced into the tunnel using a needle holder in the dominant hand, with toothed forceps in the non-dominant hand providing counter-traction. The ripcord stent suture was kept long within the tube to maintain rigidity and facilitate controlled insertion, minimising the risk of the tube catching the iris, zonules, intraocular lens haptics, or lens edge in pseudophakic eyes. The tube was advanced until the bevel was appropriately positioned, and gentle lateral movement was used to confirm freedom from iris tissue. The entry technique is demonstrated in the supplementary videos. The ripcord suture was then withdrawn and secured as required.

The tube was secured to the sclera with a 9-0 Prolene suture and covered with a pericardium or fascia lata patch graft before conjunctival closure. Subconjunctival cefuroxime and betamethasone were administered at the conclusion of the procedure. Postoperatively, patients were prescribed chloramphenicol 0.5% eye drops for four weeks and dexamethasone 0.1% eye drops, tapered weekly.

## Results

A total of 10 eyes of 10 patients were included, with a mean postoperative follow-up of 47.7 months (range, 36-57 months). Four patients had undergone previous unsuccessful trabeculectomy, and two had unsuccessful Baerveldt tube implantation before sulcus tube placement. Baseline characteristics and findings are summarised in Table 1.

**Table 1:** Baseline Characteristics and Long-Term Outcomes Following Ciliary Sulcus Glaucoma Drainage Device Implantation Baseline characteristics and long-term clinical outcomes in eyes undergoing ciliary sulcus placement of glaucoma drainage device tubes. Values represent change from baseline to final follow-up. * indicates statistical significance (p < 0.05, Wilcoxon signed-rank test). IOP = intraocular pressure; BCVA = best-corrected visual acuity; VF MD = visual field mean deviation.

Parameter	Result
Number of eyes / patients	10
Mean follow-up (months)	47.7 (range 36–57)
Lens status	3 phakic (30%); 7 pseudophakic (70%)
Prior glaucoma surgery	4 trabeculectomy (40%); 2 Baerveldt tube (20%)
Mean change in IOP (mmHg)	−14.3*
Median change in IOP-lowering medications (n)	−1.5*
Mean change in BCVA (logMAR)	−0.11
Mean change in visual field MD (dB)	+0.63

The mean preoperative intraocular pressure (IOP; Goldmann applanation) was 28.7 ± 6.9 mmHg, which reduced to 14.4 ± 2.9 mmHg at the latest follow-up. This represented a statistically significant reduction in IOP (Wilcoxon signed-rank test, p = 0.002). The median number of IOP-lowering medications decreased from three (IQR 1-4) preoperatively to 1.5 (IQR 1-3) at the latest follow-up, representing a significant reduction in medication burden (Wilcoxon signed-rank test, p = 0.014).

Best-corrected visual acuity (BCVA; logMAR) was 0.45 ± 0.47 preoperatively and 0.34 ± 0.34 at the latest follow-up, with no statistically significant change observed (Wilcoxon signed-rank test, p = 0.457).

Baseline visual field mean deviation (MD) was −14.34 ± 6.60 dB, and among eyes with paired longitudinal visual field data (n = 6), the final MD was −13.71 ± 4.66 dB. No statistically significant change in MD was detected between baseline and the last available follow-up (Wilcoxon signed-rank test, p = 0.735).

Stent suture removal was performed in six eyes, at a mean of 5.3 months postoperatively (range, 3-9 months). One eye required early postoperative paracentesis due to suspected tube obstruction by iris tissue. Pressure was controlled topically and stabilised with stent suture removal at nine months. Additional postoperative interventions included revision of tube plate position at four years, cyclodiode treatment in one eye at 18 months, and implantation of an additional glaucoma drainage device at three years for inadequate IOP control despite maximal therapy.

At the time of surgery, three eyes were phakic, and seven were pseudophakic; six eyes had undergone prior glaucoma surgery (four failed trabeculectomies and two failed Baerveldt tube implantations), while four eyes had no history of previous glaucoma surgery. One patient developed steroid-induced glaucoma due to long-term steroid use for post-laser-assisted in situ keratomileusis (LASIK) ectasia. This patient underwent a deep anterior lamellar keratoplasty (DALK) before tube surgery. The remaining patients had no documented corneal pathology before surgery. Planned stent suture removal was not classified as additional glaucoma surgery.

Additional glaucoma surgery was required in three eyes, all of which were pseudophakic with a history of prior glaucoma surgery. These comprised revision of a prominent fascia lata patch causing discomfort, cyclodiode treatment in one eye, and implantation of an additional glaucoma drainage device.

Postoperative complications were observed in three eyes. Two occurred in pseudophakic eyes with a history of prior glaucoma surgery and included intermittent intraocular pressure spikes related to poor drop compliance and corneal decompensation requiring subsequent Descemet's Stripping Automated Endothelial Keratoplasty (DSAEK). The patient who required DSAEK in 2022 had previously undergone cataract surgery in 2019, followed by implantation of a superotemporal Baerveldt tube in the anterior chamber in 2020, which subsequently failed. As the initial tube was appropriately positioned and not in contact with the corneal endothelium, it was left in situ when a second tube was inserted into the ciliary sulcus in 2021. Serial postoperative examinations confirmed that neither tube was in contact with the corneal endothelium at follow-up. The patient had a background of chronic uveitis with extensive anterior synechiae, which was considered the most likely cause of corneal decompensation. Following DSAEK, visual acuity stabilised, improving from 0.4 preoperatively to 0.2 postoperatively.

In addition, one pseudophakic eye without prior glaucoma surgery required early postoperative anterior chamber paracentesis for raised IOP. No further complications were documented in the remaining eyes without prior glaucoma surgery during follow-up.

## Discussion

In this case series, ciliary sulcus placement of glaucoma drainage device tubes was associated with sustained intraocular pressure reduction and a meaningful decrease in topical medication burden, with overall stability of visual acuity and visual field indices. Despite the close proximity of the tube to the iris and lens, no cases of pigment dispersion, persistent anterior uveitis, or cataract formation attributable to tube position were observed, supporting the intraocular safety of this approach in appropriately selected eyes.

Although postoperative complications were recorded in a small number of eyes, these occurred predominantly in pseudophakic patients with a history of prior glaucoma surgery, reflecting the complex disease profile of this subgroup rather than a consistent tube-related mechanism. Notably, corneal decompensation requiring endothelial keratoplasty occurred in one eye with multiple independent risk factors, including chronic uveitis and extensive anterior synechiae. Serial postoperative examinations confirmed that neither the anterior chamber tube nor the subsequently placed sulcus tube was in contact with the corneal endothelium, suggesting that endothelial failure was more likely related to the underlying inflammatory pathology rather than tube position. Eyes without prior glaucoma surgery experienced fewer postoperative events and did not require further surgical intervention during follow-up.

To minimise the risk of incorrect tube positioning or injury to surrounding sulcus structures, we believe that two technical steps are particularly important. First, ensuring a precise 90-degree bend in the needle used for entry provides a mechanical safeguard against inadvertent anterior misdirection. Second, deliberate alteration of the needle trajectory from an initial parallel approach to a controlled downward angulation facilitates accurate access to the ciliary sulcus. Together, these steps allow reproducible tunnel formation and stable tube positioning while reducing the risk of lens or zonular injury.

Several alternative techniques for ciliary sulcus implantation of glaucoma drainage devices have been described [5,8,12,13,14]. Many involve insertion of the tube posterior to the limbus using a straight 22-23-gauge needle under direct visualisation, while others utilise blades to create the entry site or guiding sutures to enhance control and protect adjacent intraocular structures [15]. Although effective, these methods may increase procedural complexity. In contrast, the technique described here aims to simplify sulcus entry while maintaining precision and safety and was successfully applied in both phakic and pseudophakic eyes.

Limitations

This study has several limitations. Its retrospective design, sample size of 10, and single-surgeon experience may limit the generalisability of the findings and reduce the ability to detect uncommon adverse events. In addition, the cohort largely comprised eyes with advanced or previously treated glaucoma, which reflects real-world tertiary referral practice but may not be representative of all surgical populations.

In addition, quantitative corneal endothelial cell measurements were not available, limiting assessment of subclinical endothelial changes. While clinically significant corneal decompensation was documented and tube-endothelium contact was excluded on serial slit-lamp examination, subclinical endothelial cell loss cannot be assessed without specular microscopy, limiting direct comparison with prior endothelial safety studies.

Finally, there was no control group of anterior chamber tube placements or alternative sulcus techniques. As a result, the study was not designed to provide comparative efficacy or safety data but rather to describe the feasibility, reproducibility, and long-term outcomes of a simplified sulcus entry technique.

Notwithstanding these limitations, the extended follow-up and consistent surgical approach provide meaningful insight into the feasibility and durability of this technique.

## Conclusions

In conclusion, this study demonstrates that ciliary sulcus placement of glaucoma drainage device tubes using a simplified entry technique can achieve durable intraocular pressure control with acceptable safety over long-term follow-up. The technique appears particularly useful in eyes at increased risk of corneal complications or in those without a suitable anterior chamber and offers a repeatable alternative to anterior chamber tube placement in both phakic and pseudophakic patients. Larger prospective studies are warranted to further define complication rates and comparative outcomes.

## Appendices

Materials and equipment used

The following materials and equipment were used during the surgical procedures. 

PAUL Glaucoma Implant (PGI) Manufacturer: Advanced Ophthalmic Innovations, Singapore

Baerveldt Glaucoma Implant 350 mm² (BVT-350) Manufacturer: Advanced Medical Optics (Johnson & Johnson Vision), Santa Ana, California, USA

6-0 Prolene suture Catalogue number: W8815 Manufacturer: Johnson & Johnson MedTech Ltd, Wokingham, Berkshire, United Kingdom

9-0 Prolene suture (tube fixation) Catalogue number: W1709 Manufacturer: Johnson & Johnson MedTech Ltd, Wokingham, Berkshire, United Kingdom

Supramid suture Catalogue number: 6151 Manufacturer: Advanced Medical Solutions, Winsford, Cheshire, United Kingdom

Pericardium patch graft Catalogue number: 68250 Manufacturer: Tutogen Medical GmbH, Neunkirchen am Brand, Bavaria, Germany

Fascia lata patch graft Catalogue number: 68036 Manufacturer: Tutogen Medical GmbH, Neunkirchen am Brand, Bavaria, Germany

23-gauge needle Catalogue number: 300700 Manufacturer: Becton, Dickinson S.A., Fraga, Huesca, Spain

25-gauge needle Catalogue number: 300600 Manufacturer: Becton, Dickinson S.A., Fraga, Huesca, Spain

Graefe strabismus hook Catalogue number: A2242 Manufacturer: Newcastle upon Tyne, Tyne and Wear, United Kingdom
